# Evaluation of the Long-Term Safety of Avacopan in Antineutrophil Cytoplasmic Antibody–Associated Vasculitis in the Real World (AvacoStar): Protocol for a Noninterventional Prospective Cohort Study

**DOI:** 10.2196/81415

**Published:** 2026-03-24

**Authors:** David R W Jayne, Raashid Luqmani, Benjamin Terrier, Achim Obergfell, Charlotte Pollet, Marie Boff, Monica Balcells-Oliver, Bernhard Hellmich

**Affiliations:** 1 Department of Medicine, University of Cambridge, Addenbrooke's Hospital, Hills Road, Cambridge, CB2 2QQ, United Kingdom, 44 75402000020; 2 Nuffield Department of Orthopaedics, Rheumatology and Musculoskeletal Sciences, University of Oxford, Oxford, United Kingdom; 3 Service de Médecine Interne, Hôpital Cochin, Paris, France; 4 Department of Global Medical Affairs, CSL Vifor, Glattbrugg, Switzerland; 5 Department of Biostatistics, CSL Vifor, Glattbrugg, Switzerland; 6 Medius Kliniken, University of Tübingen, Kirchheim unter Teck, Germany

**Keywords:** antineutrophil cytoplasmic antibody–associated vasculitis, ANCA-associated vasculitis, AAV, avacopan, C5a receptor, Avacopan Development in Vasculitis to Obtain Corticosteroid Elimination and Therapeutic Efficacy, ADVOCATE, granulomatosis with polyangiitis, GPA, microscopic polyangiitis, MPA, real-world evidence

## Abstract

**Background:**

Established treatments for granulomatosis with polyangiitis (GPA) or microscopic polyangiitis (MPA) include the use of immunosuppressive agents for remission induction followed by maintenance therapy. However, patients continue to experience disease progression, organ damage, and adverse events related to current therapies. Avacopan, an oral selective C5a receptor antagonist, was approved by the European Commission in January 2022 for the treatment of adult patients with severe, active GPA or MPA in combination with rituximab (RTX) or cyclophosphamide (CYC). In the pivotal phase 3 ADVOCATE (Avacopan Development in Vasculitis to Obtain Corticosteroid Elimination and Therapeutic Efficacy) study, avacopan was noninferior to prednisone taper in achieving remission at week 26 and superior in sustaining remission at week 52; furthermore, a greater improvement in estimated glomerular filtration rate with avacopan was also observed at week 52. The AvacoStar study will generate data on the benefit and risk and safety profile of avacopan in patients in a real-world context, including in those where treatment may potentially continue beyond 1 year.

**Objective:**

The primary objective of AvacoStar is to evaluate the incidence of defined medical events of special interest in patients with antineutrophil cytoplasmic antibody–associated vasculitis commencing avacopan. These include liver injury, cardiac safety, serious infections, and malignancy.

**Methods:**

AvacoStar is a noninterventional, multinational, prospective postauthorization safety study. It will enroll up to 500 patients in Germany and the United Kingdom in 2 groups of approximately 250 participants each: those treated with avacopan (plus a standard of care at local investigators’ discretion; usually an RTX- or CYC-based regimen) and a second cohort treated with a CYC- or RTX-based induction regimen without avacopan. Avacopan and the standard of care will be prescribed in the usual manner in accordance with the corresponding summary of product characteristics under the sole decision of the investigator. The treatment decision will fall within current established practice. Eligible participants will be aged ≥18 years with severe, active GPA or MPA as determined by the investigator at the time of commencing avacopan or non-avacopan standard-of-care induction therapy. Patients will be followed for up to 7 years.

**Results:**

This study has been enrolling patients since September 11, 2023. The final report is expected in the second half of 2031; interim reports are planned every 24 months after the first patient first visit.

**Conclusions:**

The AvacoStar study will be the largest European prospective real-world evidence comparative study conducted to date that evaluates the long-term safety of avacopan in severe, active GPA or MPA. This study is expected to yield important insights on the use of avacopan in severe, active GPA or MPA in a real-world setting.

## Introduction

Antineutrophil cytoplasmic antibody (ANCA)–associated vasculitis (AAV) comprises a group of rare small- and medium-sized vasculitides [1]. Granulomatosis with polyangiitis (GPA) and microscopic polyangiitis (MPA) are 2 clinicopathological forms of AAV characterized by inappropriate activation of neutrophils [2]. A third form of AAV, eosinophilic GPA, has a distinct clinical presentation and management pathway outside the scope of this study [3]. GPA and MPA are associated with the circulating ANCA against the neutrophil-expressed antigens proteinase 3 and myeloperoxidase [2].

AAV can affect several organs, but the kidneys, respiratory tract, eyes, skin, and nervous system are most commonly affected [2,3]. Despite improvements in the treatment strategies for AAV, it has been shown that long-term mortality and morbidity remain high [4]. Patients with GPA or MPA have a 1-year mortality probability of 11.1% [5]. The main causes of first-year mortality in patients with GPA or MPA are infection [5,6] and active vasculitis [5], whereas after the first year, the major causes of death are cardiovascular disease, malignancy, and infection [7,8].

Established treatments for GPA and MPA are based on the European Alliance of Associations for Rheumatology 2022, the American College of Rheumatology 2021, and the Kidney Disease: Improving Global Outcomes 2024 recommendations and involve the use of biologic and nonbiologic immunosuppressive agents for remission induction and maintenance therapy [9-11]. This consists of rituximab (RTX) and/or cyclophosphamide (CYC) with long-term use of glucocorticoids (GCs) [10,12]. Despite refinements in the dosing regimens of GCs and CYC and the introduction of RTX, patients with AAV can struggle to control their disease, with relapses occurring in 30% to 50% of patients over 5 years [13,14]. The pathology of AAV itself can lead to organ damage and chronic morbidity and mortality [15], whereas medication toxicity can also result in serious adverse effects, including increased risk of infection, malignancy, and GC-induced diabetes [13,16]. Furthermore, GCs are major contributors to impaired quality of life [17,18]. However, despite the adverse events (AEs) associated with GC therapy, GCs remain central to current induction of remission and maintenance treatment strategies [10,12].

Growing evidence has identified the role of the alternative complement cascade in the pathogenesis of AAV through activation of the C5a receptor (C5aR) [2]. Avacopan is an orally administered, small-molecule C5aR antagonist that selectively blocks the effects of C5a through the C5aR, including blocking neutrophil chemoattraction and activation [17]. Avacopan does not interfere with the production of C5b or the membrane attack complex and does not block C5a binding to a second receptor, C5L2, which is thought to have anti-inflammatory properties [19].

Avacopan was approved for the treatment of adult patients with severe, active GPA or MPA in combination with RTX or CYC by the European Commission in January 2022 [20]. The assessment of the European Union marketing authorization application for avacopan was based on data available from the clinical development program. In the phase 2 CLEAR (Complement 5a Receptor Inhibitor Levels of Efficacy and Assessment of Safety in Relapsing or Newly Diagnosed ANCA-Associated Vasculitis) study, patients treated with avacopan both with and without a reduced-dose prednisone taper achieved higher clinical response rates at 12 weeks than the control group. Both avacopan regimens demonstrated noninferiority, with AE rates remaining similar across all groups [21]. In the pivotal phase 3 ADVOCATE (Avacopan Development in Vasculitis to Obtain Corticosteroid Elimination and Therapeutic Efficacy) study, the avacopan-based regimen was noninferior to prednisone taper with the achievement of remission at week 26, was superior in sustaining remission at week 52, and was associated with improved glomerular filtration rate from baseline in patients with GPA or MPA [17]. In the ADVOCATE study, the incidences of serious AEs (SAEs), life-threatening AEs, and death were recorded. The number of SAEs (excluding events of worsening vasculitis) was 33% higher in the prednisone taper group than in the avacopan group after 52 weeks of treatment, consistent with the higher exposure to GCs in that group. In a safety analysis of combined data from the CLEAR, CLASSIC (Clinical ANCA Vasculitis Safety and Efficacy Study of Inhibitor of C5aR), and ADVOCATE clinical trials of GPA or MPA, use of avacopan was associated with fewer AEs, SAEs, white blood cell count reductions, and infections than those yielded by the non-avacopan treatment [22].

There are currently some real-world evidence studies of avacopan use that confirm ADVOCATE data in terms of remission and safety; however, cohorts are small [23,24]. In addition, the European Alliance of Associations for Rheumatology 2022 guidelines mention that long-term use of avacopan cannot be recommended at this time because there are no data on use beyond 1 year [10]. Limited data from patients treated with an avacopan-based regimen as part of an early-access program confirm the manageable safety profile of avacopan and suggest that it has the potential to be effective beyond 52 weeks in sustaining disease control [25]. Therefore, it is of interest to observe the drug in real-life use and follow up on safety in the long-term, including beyond the 12-month period of the phase 3 ADVOCATE study.

AvacoStar (NCT05897684) is a postauthorization safety study (PASS) designed to evaluate the incidence of defined medical events of special interest (MESIs) during long-term follow-up in a real-world cohort of participants commencing avacopan for severe, active GPA or MPA.

## Methods

### Study Participants and Sites

AvacoStar is a noninterventional, multinational, prospective cohort study enrolling approximately 500 patients with newly diagnosed or relapsing severe, active GPA or MPA from Germany and the United Kingdom who are receiving treatment as part of routine clinical practice. Inclusion and exclusion criteria are outlined in Textbox 1.

Textbox 1. **Study inclusion and exclusion criteria.**

**Inclusion criteria**

- Diagnosis of antineutrophil cytoplasmic antibody–associated vasculitis (AAV; granulomatosis with polyangiitis or microscopic polyangiitis) as determined by the investigator according to their usual practice
- Severe, active AAV at the time of commencing avacopan or non-avacopan standard-of-care induction therapy in the opinion of the investigator
- Participants of either sex and aged ≥18 years at the time of commencing avacopan or non-avacopan standard-of-care induction therapy
- Provision of written informed consent
- Commencement within the previous 6 months of or plans to commence avacopan, cyclophosphamide, or rituximab for the treatment of severe, active AAV outside of an interventional clinical study

**Exclusion criteria**

- Concurrent participation in an interventional study unless prospectively discussed and agreed upon with the medical monitor

### Study Design and Treatment

**Figure 1.**
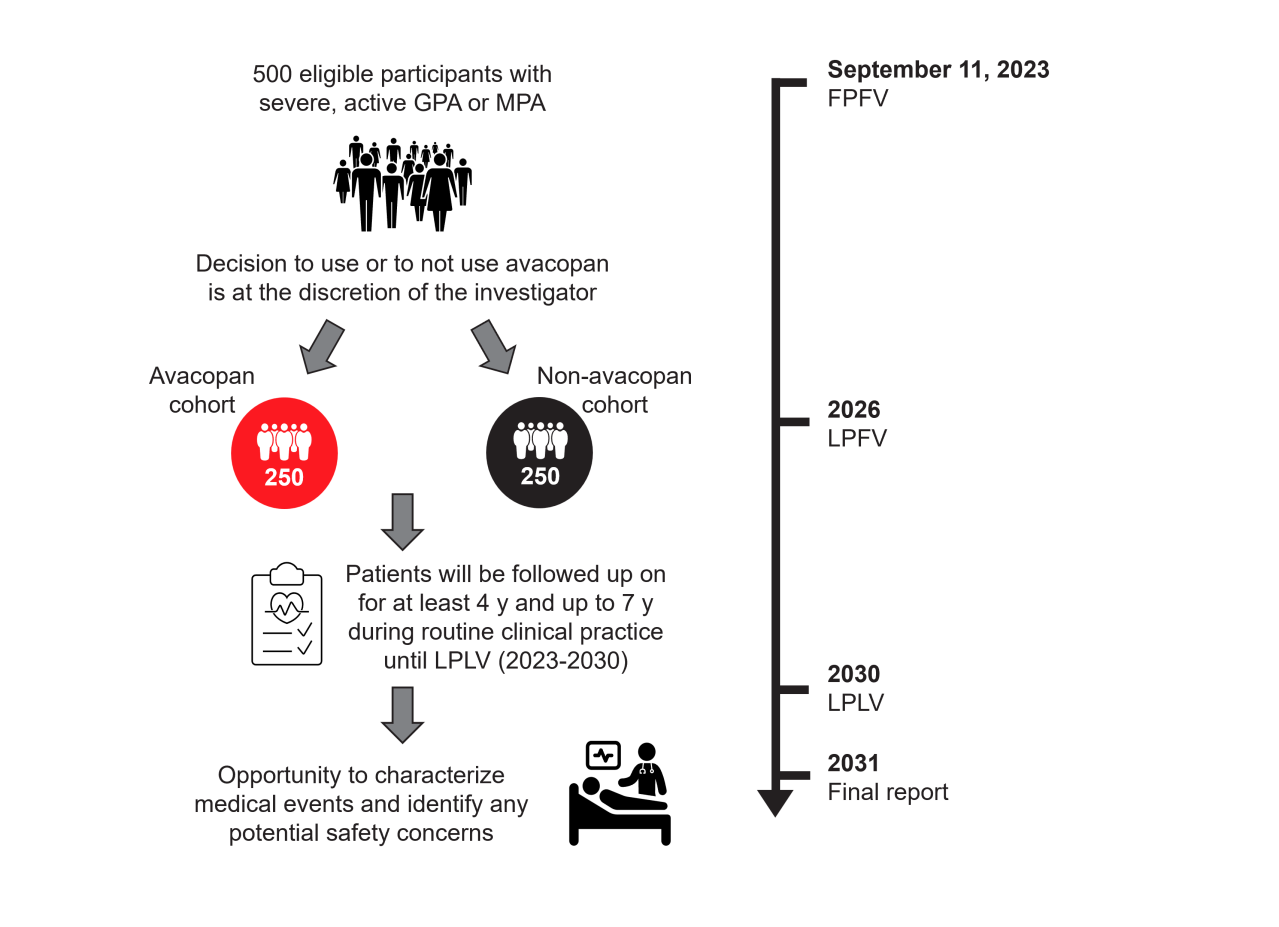
AvacoStar study design. The schematic outlines participant enrollment; treatment decision; follow-up visits; and assessment time points, including the measurement of medical events of special interest from first patient first visit (FPFV) to last patient last visit (LPLV). GPA: granulomatosis with polyangiitis; LPFV: last patient first visit; MPA: microscopic polyangiitis.

Patients are enrolled into 1 of the 2 groups: those treated with avacopan plus a standard of care at local investigators’ discretion (usually an RTX- or CYC-based regimen; the avacopan group) and a second cohort treated with a CYC- or RTX-based induction regimen without avacopan (the non-avacopan group). Avacopan and the standard of care are prescribed in the usual manner in accordance with the corresponding summary of product characteristics (SmPC) under the sole decision of the investigator. As per the SmPC, avacopan in combination with an RTX or CYC regimen is indicated for the treatment of adult patients with severe, active GPA or MPA [26]. Participants may start or stop avacopan during the study in line with usual practice. The baseline visit is defined as the day on which induction treatment (avacopan or non-avacopan standard of care with CYC or RTX) is started for active AAV. Patients are being enrolled prospectively, but up to 6 months of data may be collected retrospectively if necessary. The overall study duration is anticipated to be up to 7 years, including a recruitment period of approximately 3 years. Follow-up of enrolled patients will continue until the last patient last visit milestone, 4 years after the last participant is enrolled (Figure 1). Participants in the non-avacopan group who initiate avacopan during follow-up due to disease flare will be withdrawn from the study at that time but may cross over and re-enroll in the avacopan group if eligibility criteria are met and recruitment is still ongoing. Re-enrollment will require a new informed consent form (ICF) and assignment of a new patient identification number. Participants in the avacopan group who discontinue avacopan treatment during follow-up will remain in the study, and data will be collected as part of the avacopan group. All decisions on therapeutic or diagnostic procedures, treatments, disease management, timing of visits, and resource use are made following the investigator’s usual clinical practice. Individual participant follow-up data will be collected periodically at routine clinic visits from enrollment until the last patient last visit.

### Study Objectives

The primary objective of this study is to evaluate the incidence of defined MESIs in patients with AAV commencing avacopan. This includes the incidence (number and percentage of patients with events and total number of events), incidence per patient year, and exposure-adjusted incidence rate (EAIR) per patient year with 95% CIs of defined MESIs (liver injury, cardiac safety, serious infection, and malignancy). The secondary objectives of this study are outlined in Textbox 2. This protocol fulfills the requirements of the European Medicines Agency to conduct a PASS as a postmarketing commitment.

Textbox 2. **Secondary objectives of the AvacoStar study.**

1. To evaluate the incidence of adverse events (AEs), AEs leading to discontinuation of therapy, serious AEs (SAEs), adverse drug reactions (ADRs), serious ADRs, laboratory abnormalities, disease flares as measured using the Birmingham Vasculitis Activity Score (BVAS), and organ damage as measured using the Vasculitis Damage Index (VDI) in patients with antineutrophil cytoplasmic antibody–associated vasculitis (AAV) commencing avacopan
2. To evaluate the background incidence of AEs, medical events of special interest (MESIs), SAEs, laboratory abnormalities, disease flares as measured using the BVAS, and organ damage as measured using the VDI in a similar population of patients with severe, active AAV but not receiving avacopan
3. To compare SAEs and MESIs in patients with AAV between patients treated with and without avacopan
4. To describe patterns of immunosuppression and glucocorticoid use in a real-world cohort
5. To describe avacopan use in a real-world cohort

### Statistical Analysis

Statistical analyses will be exploratory in nature, performed using the SAS statistical software (version 9.4 or later; SAS Institute). All variables will be analyzed descriptively using appropriate statistical methods: categorical variables will be analyzed via frequency tables (absolute and relative frequencies), and continuous variables will be analyzed via descriptive statistics (ie, number of patients, mean, SD, minimum, median, quartiles, and maximum). Continuous variables will be summarized via absolute value and changes from baseline per analysis time point if applicable. Where appropriate, 95% CIs will be provided. Comparisons between treatment groups will be performed using appropriate methods to adjust for potential bias. A propensity score will be constructed by estimating the probability of exposure by regressing the treatment assignment on baseline characteristics using logistic regression. Treatment group comparisons will be conducted using 2 methods: adjustment (the propensity score is included in the regression model) and inverse probability weighting. The proportion of patients with at least 1 event will be analyzed using logistic regression (using the Firth correction if the event is rare), and results will be reported as odds ratios with 95% CIs and 2-sided *P* values, along with estimated event proportions (least squares mean) and their 95% CIs. Incidence rates (IRs; unique IR, EAIR, and unique EAIR) per patient year will be analyzed using negative binomial regression (SAS GENMOD), with results reported as rate ratios with 95% CIs and 2-sided *P* values, along with the corresponding estimated rates per patient year and their 95% CIs. Outcomes of statistical comparisons will be interpreted with caution.

### Sample Size and Power Calculations

The sample size of 250 participants in the avacopan group was considered reasonable; given an expected attrition rate of 15% or 10% per year, the power of the study to detect a single event will be 77% or 81%, respectively, for an AE with an IR per year of 0.2% for a follow-up duration of 4 years. Malignancy related to immunosuppression is the most infrequent form of the expected AEs, with the literature reporting an incidence of 1.4% per year. A sample size sufficient to detect an AE with an IR of 0.2% per year is well placed to detect a malignancy event related to immunosuppression. Skin cancer is the most common malignancy related to immunosuppression after organ transplantation, with a US study reporting a 1.4% annual incidence, well within the detection limit of this study [27].

### Analysis of Primary End Point

For MESIs, the incidence (number and percentage of patients with events and total number of events), as well as the incidence per patient year, will be provided for the avacopan group. For each MESI, both the IR and EAIR will be calculated with CIs. The EAIR will account for individual differences in patient follow-ups. The EAIR per patient year will be calculated as (number of MESIs)/(total patient years of exposure). Total patient years of exposure is calculated as the sum of each patient’s individual treatment duration measured from treatment initiation to the end of the exposure period. The incidence of MESIs will be presented by year as well as cumulatively over the full period of observation. To determine whether MESIs occurring after avacopan has been discontinued should be reported as part of the avacopan group, appropriate risk windows will be used. Malignancy events will be analyzed using an indefinite risk window irrespective of last avacopan dose, whereas other events (liver injury, cardiac safety, and serious infections) will be analyzed using a risk window of 9 weeks at either the end of follow-up or last avacopan dose.

### Analysis of Secondary End Points

IRs of AEs leading to discontinuation of therapy, serious adverse drug reactions, and adverse drug reactions for the avacopan group will be presented. For AEs, SAEs, and MESIs, the incidence as well as the incidence per patient year will be provided by treatment group, overall, and by Medical Dictionary for Regulatory Activities system organ classes and preferred terms. All AEs and treatment-emergent AEs will be reported separately. For SAEs and MESIs, treatment groups will be compared for the proportion of patients with at least 1 event and the number of events per year, and for the IRs per patient year, the unique IR, EAIR, and unique EAIR will be reported. Descriptive statistics will also be provided by treatment group for change in Vasculitis Damage Index (VDI), change in laboratory assessments over time, proportion of GC-free patients over time, concomitant and cumulative immunosuppression over time, incidence of disease flares, and time to first flare as assessed using the Birmingham Vasculitis Activity Score (BVAS) for patients achieving remission. The duration of treatment with avacopan by reason for treatment discontinuation will be summarized descriptively.

### Assessments

Baseline data being collected include demographics; smoking history; MPA or GPA diagnosis; medical history and comorbidities; prior treatment with immunosuppressive drugs; treatment group and exposure; investigator’s reason for choosing the treatment group; ANCA titers; and laboratory values, BVAS, and VDI. Variables for each of the treatment groups being collected during the follow-up period include information on the use of immunosuppressives alongside avacopan- and non–avacopan-based regimens, ANCA titers, laboratory values, BVAS, VDI, AEs, MESIs, SAEs, selected safety laboratory tests, disease-related damage, flares, and use of concomitant immunosuppression and GCs. Data on AEs observed from baseline, including the seriousness, timing of onset, duration, causal relationship to avacopan, action taken with regard to avacopan administration and dosing in response to the event, and outcome of the event will also be collected. The expected timings for the collection of each variable are presented in Table 1. The study team will ensure data quality through targeted on-site verification of critical variables to confirm accuracy against source documents. Remote monitoring will enable continuous oversight, timely query resolution, and early detection of inconsistencies and deviations from the protocol. Periodic data review and ongoing data cleaning will further ensure that the final dataset is complete, reliable, and fit for analysis.

**Table 1.** Planned study schedule and expected timing for the collection of each variable in the AvacoStar study.

Frequency of measurements	Specific measurement variable
At enrollment	Signed informed consent form^a^ Inclusion and exclusion criteria Year of birth, sex, ethnicity, and race
At baseline^b^^,c^	Height and body weight Smoking history and current status MPA^d^ or GPA^e^ diagnosis^f^ Medical history and comorbidities^g^ Prior treatment with immunosuppressive drugs^h^ Investigator’s reason for choosing the treatment group ANCA^i^ binding levels^j^
At baseline, every 3 months for the first year^c^, and every 6 months after the first year	Treatment group and exposure^k^
Every 3 months for the first year, every 6 months after the first year, and at study exit or end of study	ANCA positivity^l^ Treatment discontinuation and reasons
At baseline, every 3 months for the first year, every 6 months after the first year, and at study exit or end of study	Laboratory tests^m^ BVAS^n^ (version 3.0)^o^ VDI^p^ Concomitant medication^q^ Recording of AEs^r^, SAEs^s^, ADRs^t^, SADRs^u^, and MESIs^v^ (liver injury, serious infections, malignancy, and serious cardiac events)^w^
At study exit or end of study	Study discontinuation and reasons

a Informed consent includes the approval to collect retrospective data.

b Baseline visit corresponds to avacopan or non-avacopan induction therapy (cyclophosphamide or rituximab) start date.

c Patients who started avacopan or standard of care within 6 months of study start may have these data collected retrospectively.

d MPA: microscopic polyangiitis.

e GPA: granulomatosis with polyangiitis.

f Including antineutrophil cytoplasmic antibody–associated vasculitis phenotype, year of diagnosis, pattern of organ involvement, year of last relapse, number of relapses in the past 5 years, history of positive antineutrophil cytoplasmic antibody titer, and specificity.

g Medical history includes etiology of underlying disease and history of major related health issues and procedures (eg, surgery due to underlying disease).

h Including dose, duration before start of treatment, and reason for change in dose or medication.

i ANCA: antineutrophil cytoplasmic antibody.

j At baseline, if the patient is ANCA positive, proteinase 3 and/or myeloperoxidase binding levels will be collected (if available). The highest proteinase 3 and myeloperoxidase titers in the patient medical history will be collected (if available).

k Including start date; stop date (if applicable); daily dose or frequency; route of administration (intravenous or oral); reason for treatment initiation or switch; and rationale for starting, stopping, or changing the dose (completion of planned course, adverse event, investigator’s or patient’s decision, withdrawal of consent, administrative problems, death, or other).

l During follow-up, proteinase 3 or myeloperoxidase positivity will be collected.

m Includes creatinine, estimated glomerular filtration rate, immunoglobulin G, creatine phosphokinase, alanine transaminase, aspartate transaminase, bilirubin, white blood cell count, and albumin if collected per the site’s routine practice.

n BVAS: Birmingham Vasculitis Activity Score.

o During the follow-up, the BVAS will be collected at each flare episode.

p VDI: Vasculitis Damage Index. At baseline, the VDI is modified to permit a baseline assessment that captures prior (non–vasculitis-related) damage, including infections.

q Including start date; stop date (if applicable); daily dose or frequency; route of administration (intravenous or oral); reason for treatment initiation or switch; and rationale for starting, stopping, or changing the dose (completion of planned course, adverse event, investigator’s or patient’s decision, withdrawal of consent, administrative problems, or other). Limited to glucocorticoids and immunosuppressants.

r AE: adverse event.

s SAE: serious AE.

t ADR: adverse drug reaction.

u SADR: serious ADR.

v MESI: medical event of special interest.

w AEs, SAEs, ADRs, or MESIs will be collected from baseline up to 9 weeks after the end of follow-up. Collection of safety events includes the seriousness, duration, causal relationship to avacopan, action taken with avacopan in response to the event, and outcome of the event.

### Ethical Considerations

This study is being conducted within an approved indication in accordance with the Declaration of Helsinki, the International Society for Pharmacoepidemiology Guidelines for Good Publication Practice, the European Medicines Agency guideline on good pharmacovigilance practices module VI and VIII for PASSs, and local laws and regulations as applicable in each participating country [28-30]. The PASS will comply with Revision 6 of the European Network of Centres for Pharmacoepidemiology and Pharmacovigilance methodological standards for study protocols, the European Network of Centres for Pharmacoepidemiology and Pharmacovigilance Checklist for Study Protocols, and the guidelines for good publication practice of the International Society for Pharmacoepidemiology, as well as the data vendor’s (IQVIA) quality management system [31-33]. Additionally, guidelines of the International Council for Harmonisation of Technical Requirements for Pharmaceuticals for Human Use concerning good clinical practice are being followed whenever possible [34].

Each study site has received approval from their regulatory authority or ethics committee before study initiation. A full list of the ethics committees and application IDs is available in Multimedia Appendix 1.

Written informed consent must be obtained from the patient (or their legally authorized representative) before participation in the study and in accordance with the local requirements. The informed consent process and signed ICF must be documented and included in their medical file, and a copy of the signed ICF (with certified translation if applicable) must be provided to the patient or representative. All signed and dated ICFs must be retained in the study file and be available for monitoring at any time. Patients may withdraw consent at any time without it affecting their medical care or access to treatment. Participants in the non-avacopan group who initiate avacopan during follow-up may re-enroll in the avacopan group if eligibility criteria are met and recruitment remains open, and they must resign the ICF at re-enrollment. Patients will not receive any compensation for their participation in this study.

Patient data are collected in a pseudonymized or deidentified form. Any new information that might influence the evaluation of the benefit and risk balance of the product shall be immediately communicated to competent authorities of member states. The analysis will be performed on the pseudonymized or deidentified patient-level dataset. The data system will be maintained and secured as required by applicable laws and regulations. Processes assuring data security will be used during data extraction, storage, and backup. The progress reports and final analysis will be reviewed and evaluated by a medical monitor and/or pharmacovigilance delegate.

## Results

This study has been enrolling patients since study start on September 11, 2023, with 35 sites actively participating in enrolling patients. The final report is expected in the second half of 2031. Interim reports are planned every 2 years from the start of enrollment and will provide information about the progress of the study. All enrollment has now been completed; data analysis is under way, and the first interim report will be submitted to the European Medicines Agency in March 2026.

## Discussion

### Overview

Despite current treatments for AAV, disease burden still has a high impact on the quality of life of individuals with GPA or MPA [17,18,35], and many do not achieve or sustain remission [36,37]. Existing treatments cannot induce sustained disease control in all patients, and immunosuppressive agents, including GCs, are associated with frequent clinically relevant AEs and comorbidities, both resulting in impaired quality of life [13,15]. There is an unmet need for alternative, more targeted, and less toxic therapeutic options [38].

The avacopan-based regimen in combination with RTX or CYC may be considered for induction of remission in AAV as part of a strategy to substantially reduce exposure to a tapering prednisone regimen [10,11,17,39]. However, the safety of avacopan beyond 52 weeks has not been assessed. Therefore, it is of interest to monitor the drug in daily use as per clinical practice and generate long-term safety data in real-world settings. This information will be gathered in the context of this study. The main rationale for this PASS is to further understand the identified and potential risks of avacopan described above by studying the use of avacopan among patients in a real-world context, where treatment may extend beyond 1 year. The AvacoStar study is designed to assess long-term safety and observe avacopan in daily use in a real-world context.

### Study Design Features

Given the heterogeneous clinical presentation of patients with AAV, the planned multicenter, multinational large sample size will provide an adequate representative number of patients. The data collected in this study will be frequently monitored during the study lifetime. Data collection will ensure that applicable variables of interest and critical data elements are routinely collected as part of real-world clinical care and are available via medical charts and physician reporting.

### Strengths of the Study Design

To our knowledge, this is the largest European prospective real-world evidence study of avacopan in patients with GPA or MPA. This study evaluates long-term safety data on the use of avacopan and is the longest study to date. This study will also generate data that reflect real-world management of AAV. Although we are recruiting from a European population, the multicenter, multinational study design will facilitate the generalizability of the study results.

### Limitations of the Research Methods

As this is a noninterventional study, there are several potential confounding factors to be considered. Although several steps were taken to minimize bias, it is likely that some residual bias remains and, therefore, the results of any direct comparisons between the avacopan and non-avacopan groups will need to be interpreted with this in mind. For example, avacopan may be used by clinicians preferentially in patient subgroups where randomized controlled trial data have shown the greatest benefit (eg, severe, active renal disease or presence of GC-related comorbidities). Eligibility criteria have been selected to ensure that baseline characteristics in the non-avacopan group are similar to those of the avacopan group, and all participants enrolled in the study will be potentially eligible to receive avacopan according to the European SmPC even if they are enrolled into the non-avacopan group. Additionally, detailed information will be collected at baseline on potential confounding factors, including those related to disease severity, diagnosis, prior immunosuppression exposure, and MESI-related risk factors. To account for the risk of bias resulting from confounding factors, treatment groups will be compared using propensity scoring methodology.

### Conclusions

The AvacoStar study will be a large, prospective real-world evidence study that evaluates the long-term safety of avacopan in GPA or MPA. The study is expected to yield important insights on the use of avacopan in GPA or MPA in a real-world setting, including treatment beyond 12 months.

## Supplementary material

10.2196/81415 Multimedia Appendix 1 Full list of ethics committees and application IDs.

## Abbreviations

AAV: antineutrophil cytoplasmic antibody–associated vasculitis
ADVOCATE: Avacopan Development in Vasculitis to Obtain Corticosteroid Elimination and Therapeutic Efficacy
AE: adverse event
ANCA: antineutrophil cytoplasmic antibody
BVAS: Birmingham Vasculitis Activity Score
C5aR: C5a receptor
CLASSIC: Clinical Antineutrophil Cytoplasmic Antibody Vasculitis Safety and Efficacy Study of Inhibitor of C5a Receptor
CLEAR: Complement 5a Receptor Inhibitor Levels of Efficacy and Assessment of Safety in Relapsing or Newly Diagnosed Antineutrophil Cytoplasmic Antibody–Associated Vasculitis
CYC: cyclophosphamide
EAIR: exposure-adjusted incidence rate
GC: glucocorticoid
GPA: granulomatosis with polyangiitis
ICF: informed consent form
IR: incidence rate
MESI: medical event of special interest
MPA: microscopic polyangiitis
PASS: postauthorization safety study
RTX: rituximab
SAE: serious adverse event
SmPC: summary of product characteristics
VDI: Vasculitis Damage Index
