# Supplementary material for: Evaluation of the Long-Term Safety of Avacopan in Antineutrophil Cytoplasmic Antibody–Associated Vasculitis in the Real World (AvacoStar): Protocol for a Noninterventional Prospective Cohort Study
Source: JMIR Res Protoc. 2026 Mar 24;15:e81415. doi: 10.2196/81415 (PMC13012226; doi:10.2196/81415)
Supplement: Multimedia Appendix 1 [file resprot-v15-e81415-s001.docx]

**Multimedia Appendix 1**

This appendix has been provided by the authors to give readers additional information about their work.

**Supplementary table 1.** Full list of regulatory authorities and ethics committee along with application IDs for all sites participating in the AvacoStar study.

| Country |  | Ethical committee or institutional review board | Application ID | Approval date |
| --- | --- | --- | --- | --- |
| Germany | Regulatory Authority | Bundesinstitut für Arzneimittel und Medizinprodukte (BOPST) | 7746 | 01 Sep 2023 |
|  | Central ethics committee | Landesaerztekammer Baden-Wuerttemberg | F-2023-077 | 25 Aug 2023 |
|  | Local ethics committee | Ethikkommission Fakultata der Universitat Duisburg-Essen | 23-11485-BO | 07 Feb 2024 |
|  |  | Ärztekammer Westfalen Lippe | 2023-592-b-S | 27 Oct 2023 |
|  |  | Local Ethikkommission der Univerität Köln | 23-1344_1-NIS | 29 Jan 2024 |
|  |  | Sächsische Landesärztekammer Ethikkommission-LEC | EK-BR-106/23-1 | 24 Oct 2023 |
|  |  | Ethikkommission der Aerztekammer Hamburg | 2023-200818-BO-bet | 26 Sep 2023 |
|  |  | Ethik-Kommission der Aerztekammer Niedersachsen-LEC | Grae/107/2023 | 18 Sep 2023 |
|  |  | Ethik-Kommission der Albert-Ludwigs-Universität Freiburg | 23-1447_1-S1-AV | 19 Dec 2023 |
|  |  | Charité - Campus Virchow-Klinikum | 23-0709 | 01 Dec 2023 |
|  |  | Ethik-Kommission an der Medizinischen Fakultät der Universität | 23-0773 | 19 Mar 2024 |
|  |  | Ethik-Kommisssion der Medizinischen Fakultaet der Georg-August-Universitaet Goettingen | 24/10/23 Ü | 11 Oct 2023 |
|  |  | Ethik-Kommission der Ärztekammer Westfalen-Lippe und der Med. Fakultät der Universität Münster | 2023-592-b-S | 17 Apr 2024 |
| United Kingdom | Central ethics committee | NRES Committee London - Brent | 318568 | 24 Jul 2023 |
